# Productive Performance, Milk Composition and Milk Fatty Acids of Goats Supplemented with Sunflower and Linseed Whole Seeds in Grass Silage-Based Diets

**DOI:** 10.3390/ani10071143

**Published:** 2020-07-06

**Authors:** Einar Vargas-Bello-Pérez, Carlos Alberto García Montes de Oca, Nazario Pescador Salas, Julieta G. Estrada Flores, José Romero Bernal, Lizbeth Esmeralda Robles-Jimenez, Manuel Gonzalez-Ronquillo

**Affiliations:** 1 Department of Veterinary and Animal Sciences, Faculty of Health and Medical Sciences, University of Copenhagen, Grønnegardsvej 3, DK-1870 Frederiksberg C, Denmark; 2 Universidad Autónoma del Estado de México, Facultad de Medicina Veterinaria y Zootecnia, Instituto Literario 100 Ote., 50000 Toluca, Estado de México, Mexico; pollovet@yahoo.com.mx (C.A.G.M.d.O.); npescadors@uaemex.mx (N.P.S.); gazapo79@yahoo.com.mx (J.R.B.); lizroblez@hotmail.com (L.E.R.-J.); 3 Universidad Autónoma del Estado de México, Instituto en Ciencias Agropecuarias y Rurales, Instituto Literario 100 Ote., 50000 Toluca, Estado de México, Mexico; jgestradaf@uaemex.mx

**Keywords:** dairy goats, fatty acids, human health, linseed, sunflower, milk fat

## Abstract

**Simple Summary:**

Feeding is the most practical way to induce changes in the milk fatty acid profile of ruminants. The use of whole oilseeds is an available resource that farmers can use for improving animal´s performance and milk quality. Thus, the objective of this study was to determine productive performance, milk composition and milk fatty acids of goats supplemented with sunflower and linseed whole seeds in grass silage-based diets. Compared to the typical use of Megalac-R^®^, sunflower and linseed whole seeds did not affect nutrients digestibility and milk composition but reduced palmitic acid and increased oleic acid in milk. Overall, sunflower and linseed whole seeds resulted an effective strategy for altering the fatty acid composition of goat´s milk towards a healthier profile for humans without negative effects on animal performance.

**Abstract:**

The objective of this study was to determine productive performance, milk composition and milk fatty acids (FA) of goats supplemented with sunflower and linseed whole seeds in grass silage-based diets. Nine Alpine goats were grouped in a replicated 3 × 3 Latin square design (n = 3), that included three 21-d periods. Treatments were based on grass silage offered *ad libitum* and a concentrate mixture supplemented with either 40 g/d of Megalac-R^®^ (control), 80 g/d of sunflower seed (SF), or 80 g/d of linseed (LS). Dry matter intake (1292 ± 14.0 g/d) and digestibility (g/kg) of dry matter (640 ± 32.1), organic matter (668 ± 32.4), neutral detergent fiber (628 ± 41.4) and acid detergent fiber (567 ± 60.9) was not affected by treatments (*p* > 0.05). Treatment did not affect milk fat yield (39.9 ± 1.24 g/d), protein content (4.5 ± 0.03 %) and protein yield (34.7 ± 1.22 g/d). Compared to control, SF and LS, decreased C16:0 (28.2 vs. 23.1 and 22.4 g/100 g), and increased total C18:1 (24.1 vs. 27.6 and 28.4 g/100 g) respectively. Overall, SF and LS resulted an effective strategy for altering the FA composition of goat´s milk towards a healthier profile for humans without deleterious effects on animal performance.

## 1. Introduction

Milk fat is an important component of the nutritional quality of goat dairy products [1]. Some milk fatty acids (FA) such as oleic and linolenic acids have been shown to exert positive effects on human health as they promote a direct vascular antiatherogenic effect [2]. In addition, goat’s milk fat is more digestible than bovine milk fat and this may be related to the lower milk fat globules size, and the higher contents of short- and medium-chain FA in goat’s milk [3]. Goat milk fat content and composition can be extensively modified by genetic and nutritional factors [4]. Dietary fat supplementation is an efficient nutritional strategy to modify milk FA composition in lactating ruminants that could be used to improve the nutritional quality of milk fat [5].

Dietary factors such as forage type and feeding system affects goat’s milk FA composition [6]. Conserved forages can also provoke differential changes in milk FA [7]. For example, wilting during haymaking is associated with decreases in grass polyunsaturated FA concentrations (such as C18:3n-3) due to oxidative losses [8]. This can result in lower concentrations (up to 75%) of C18:3n-3 in hay compared to grass silage [9]. Additionally, grass is generally harvested later for making hay than silage, which could interact as an effect of the physiological stage of grass to decrease hay C18:3n-3. 

Experiments with lactating cows have established that the forage source in the diet is a major determinant of milk fat composition as well as dietary oilseeds rich in unsaturated FA (i.e., rapeseed, linseed, sunflower) [10]. In dairy goats, the use of oilseeds such as sunflower and linseed, has been an effective nutritional strategy that in some cases improve feed intake, milk yield and/or protein and fat content and therefore, economic profitability for the farmers [7]. In addition, the use of rumen-protected fats rich in polyunsaturated FA has been shown to improve milk, fat and protein yields in dairy goats [11].

Until now, it is unknown if feeding goats with rumen-protected fats improves milk FA composition as compared to whole oilseeds. Thus, the objective of this study was to determine productive performance, milk composition and milk (FA) of goats supplemented with rumen-protected (Megalac-R^®^) or unprotected (whole seeds of sunflower and linseed) unsaturated FA sources in grass silage-based diets. We hypothesized that compared to protected fats, unsaturated FA sources supplied as whole oilseeds in goat diets will promote a healthier FA profile for human consumption without deleterious effects on production performance. Results from this study will orientate farmers for the best choice of dietary fat for lactating goats.

## 2. Materials and Methods

### 2.1. Animals and Diets

All experimental procedures were approved by the Animal Experimental Guidelines of the Universidad Autonoma del Estado de México (project code UAEMex2633/2008U). Nine multiparous Alpine goats (50.8 ± 5 kg BW), in mid-lactation (70 ± 3 days) were offered three diets according to a replicated 3 × 3 Latin square design (n = 3) with 21 d experimental periods using three animals per treatment. Each experimental period comprised of 15-d adaptation and 6-d sampling periods. Goats were housed in individual stalls, with continuous access to water and milked daily at 08.00 hours. 

Treatments were based on grass hay and grass silage offered *ad libitum* and a concentrate mixture (Table 1) supplemented with either 40 g/d of Megalac-R^®^ (control), 80 g/d of sunflower seed (SF), or 80 g/d of linseed (LS) (Table 2). The control treatment was composed (% of dry matter) by 20% of grass silage, 38% of grass hay, 30% of sorghum grain, 7% of canola meal, 3% of Megalac-R^®^, and 2% of minerals. SF was composed (% of dry matter) by 20% of grass silage, 37% of grass hay, 33% of sorghum grain, 2% of canola meal, 6% of sunflower seed, and 2% of minerals. LS was composed (% of dry matter) by 20% of grass silage, 39% of grass hay, 32% of sorghum grain, 1% of canola meal, 6% of whole linseed, and 2% of minerals. The forage to concentrate ratios for experimental treatments was: 58:42 for control, 57:43 for SF and 59:41 for LS. Diets were formulated to meet energy and protein requirements [12]. Diets were formulated to contain 115 g/kg CP and 2.40 Mcal ME/kg DM [13]. 

The concentrate was offered twice a day at 08.00 and 16.00 hours. Depending on the treatment, each animal received daily the following amounts of concentrate: 710 g for control, 624 g for SF and 596 g for LS. The concentrate and forages were offered separately as each individual stall had a feed bunk divided in two spaces. 

For hay and silage, a ryegrass meadow with clover was used. A cut was made every 35 days at a height of 5 cm from the ground level. Of the total cut, half was used to make silage in 50 kg plastic bags. Plant material was placed in layers, compacted, sealed and ensiled for 21 days. The other half of the grass material was sundried until it reached approximately 90% dry matter and was stored as square hay bales in a ventilated barn. The concentrate was made in batches of 100 kg by manually mixing the ingredients for each experimental diet.

### 2.2. Sampling and Measurements

Individual body weights (BW) were measured at the beginning and end of each experimental period. Individual dry matter (DM) intake was recorded daily, but only measurements collected during the last six days of each experimental period were used for statistical analysis. During each experimental period, samples of hay, grass silage and concentrates were composited daily and stored at −20 °C. Feces were collected at 08.00 hours on the last six days of each experimental period. For feces collection, each individual stall was equipped with a metallic container with a mesh frame. Feces samples were fully collected and used for calculations. A subsample of 10% was taken for analysis. Diets, orts and fecal samples were dried in a forced-air oven at 60 °C for 48 h. Once dried they were ground with a Wiley mill (model 3, 2.0 mm screen; Arthur H. Thomas, Philadelphia, PA, USA), and analyzed for DM, organic matter (OM) and nitrogen (N) using the AOAC [14] methods 930.15, 942.05 and 990.02, respectively. 

Urine samples were collected daily at 08:00 h during the last 6 days of the experimental periods. Urine was collected using a metal container under each individual stall. Total volume of urine per day was measured and subsequently 10% of total samples was collected and frozen at −20 °C for further analysis. Feces and urine samples were used to determine N intake and excretion. For feces, the CP content was calculated by multiplying the N content by 6.25. Neutral detergent fiber (NDF), acid detergent fiber (ADF) and acid detergent lignin (ADL) were determined following Van Soest et al., [15] methods with alpha amylase and uncorrected for ash. Nutrient digestibility (g/kg) was determined as ((nutrient intake, g/d–nutrient excreted, g/d) / (nutrient intake, g/d)) × 1000. 

Body weight (kg) and body weight change (BWC, g/d) were recorded during the beginning and at the end of each experimental period. Dry matter intake (DMI, g/d) and individual milk yields (kg/d) were recorded daily. Milk yield was recorded on the last 6 days of each experimental period, collected at 08.00 hours using a volumetric milk meter and were considered for statistical analysis. Samples were preserved with potassium dichromate (Merck, Fontenay-Sous-Bois, France). Milk samples were analyzed by infrared Ecomilk Milk (Ekomilk-M, Milkana KAM 98-2A, Hillerød, Denmark) to obtain the values of protein, fat, total solids (TS) and non-fat solids (NFS). Milk urea nitrogen (MUN) was analyzed using the Micro-Kjeldahl method, and the protein content was calculated as the nitrogen percentage multiplied by 6.38 based on method 991.20 [14]. For FA analysis, unpreserved milk samples were collected during the last 6 days of each experimental period and stored at −20°C until analysis. 

Fat-corrected milk (FCM) was calculated at 3.5%, FCM (kg/d) = (milk (kg/d) × 0.432) + (fat (kg/d) × 16.216]), energy corrected milk (ECM) was calculated as, ECM = (milk (kg/d) × 0.327) + (fat (kg/d) × 12.86) + (protein (kg/d) × 7.65) [16]. The feed efficiency (FE) was calculated using the following formula: FE = milk yield (kg/d)/dry matter intake (kg/d). Adjusted FE was calculated using the following formula = 3.5% FCM (kg/d)/dry matter intake (kg/d).

### 2.3. Fatty Acid Analysis

Lipids in 130 mg of lyophilized milk samples were extracted in 10 mL of a mixture of hexane–diethyl ether (50:50, v/v), 1 mL saturated NaCl solution and 1 mL ethanol. After mixing, the organic phase was recovered by centrifugation at 1000 rpm for 10 min at 48°C, repeatedly rinsed (n = 3) with 5 mL of a mixture of hexane–diethyl ether (50:50, v/v) and dried under N_2_. Fatty acid methyl esters (FAME) were prepared following the addition of 100 mL of 1M-sodium methanolate at room temperature for 10 min followed by 500 mL of 14% (v/v) boron trifluoride in methanol for 10 min [17]. 

Methyl esters were quantified by GLC using a gas chromatograph (Claurus 500, PerkinElmer, Waltham, MA, USA) equipped with autosampler and a flame-ionization detector and 100m fused silica capillary column (CP-SIL 88; Chrompack 7489, Middelburg, The Netherlands) using H_2_ as the carrier and fuel gas. Total fatty acid methyl esters (FAME) profile in a 2-mL sample at a split ratio of 1:50 was determined using a temperature gradient program [18]. Fatty acid GC peaks were identified by using a FA methyl ester standard (FAME; Supelco 37 Component FAME mix, Bellefonte, PA, USA).

### 2.4. Statistical Analysis

Unless otherwise specified, averaged data from the last 6 days of each experimental period were used for statistical analysis. Data were subjected to ANOVA using the general linear model procedure of the SAS software package [19] with a model that included the effects of goat, period, and treatment in a Latin square design:

Y_ijk_ = μ + D_i_ + A_j_ + P_k_ + ε_ijk_ (1)

where μ is the mean, D_i_ is the effect due to the diet, A_j_ is the animal effect, P_k_ is the effect due to the experimental period, and ε_ijk_ is the experimental error. Fixed effects were experimental period and treatment, and the random effect was individual goat. Least-square means with their standard errors are reported and treatment effects were declared significant at *p* < 0.05.

## 3. Results and Discussion

### 3.1. Diet Composition (Individual Feedstuffs and Treatment Diets)

Only numerical differences in the chemical composition of individual feedstuffs and dietary treatments are reported. In terms of individual feedstuff’s chemical composition, DM contents for grass hay and grass silage were expected (Table 1). Interestingly, contents for NDF from linseed and sunflower seeds were almost in the same magnitude as those from hay and silage. Linolenic acid was the most predominant fatty acid in whole linseed. Sunflower seed was mostly composed by oleic and linoleic acids whereas Megalac-R^®^ was composed by palmitic and oleic acids. Regarding each treatment’s chemical composition, as initially formulated, protein and energy contents were similar between treatments. Compared to control diet, ether extract from SF and LS was slightly lower than control. One could expect ether extract to be higher in those diets with oilseed-by products, however, one possible explanation is that canola meal inclusion was higher for control in order to formulate iso-energentic and iso-proteic diets.

Compared to control, SF was characterized by higher contents of oleic and linoleic acids and LS by its higher contents of linoleic and linolenic acids (Table 2). Analyzing individual feedstuffs and dietary treatments is important as it provides a wider picture of what animals eat and how the animals use nutrients. In this study, the inclusion of oilseed is relevant as they provided dietary protein, ether extract and neural detergent fiber.

### 3.2. Animal Performance (Intake and Digestibility)

Goat’s BW did not differ between the groups along the trial and no oil supplement refusals were found. Total DM intake tended (*p* = 0.06) to increase (Table 3) with SF and LS. OM intake was lower (*p* < 0.05) for control and LS compared with SF; NDF and ADF intakes were lower for control compared with LS and SF. Similarly, when lactating goats [20], lactating ewes [21], and dairy cows [22], were fed with diets supplemented with sunflower oil or linseed, no differences in DM intake were found. 

Grass silage intakes were not affected by treatments (*p* > 0.05), however, the grass hay intake was higher (*p* < 0.05) for LS diet compared with control and SF diets. On the contrary concentrate intake was higher for SF diet (*p* < 0.05) followed by control and LS diets. Although fat intake (g/d) was higher for control (*p* < 0.05), followed by SF and LS, it is possible that the amount of supplemental FA sources was enough to promote shifts in the rumen biohydrogenation pathways and later this was reflected in reduced milk concentrations of C16:0 and C18:2 in LS compared to that of control.

Total digestibility (g/kg) for DM (641), OM (668), NDF (629), ADF (567) and ADL (375) were similar between treatments. The lack of differences on nutrients digestibility from dietary treatments somehow indicated that the treatment formulations were appropriate. This is similar to Haro et al., [23] who did not find differences in digestibility in lambs fed with sunflower seed or sunflower meal. However, others have reported changes in the NDF digestibility when goats are fed with oilseed by-products. For example, Silva et al. [24] observed no differences in NDF digestibility at different inclusion levels of soybean oil in dairy goat diets, whereas Karalazos et al., [25] observed that NDF digestibility increased in diets containing 17%, 35% and 53% of cottonseed inclusion when compared to the control treatment. The discrepancies between similar studies and our results may be related to the differences in the composition of the concentrate used in the different trials. 

Nitrogen intake was higher in control, compared to SF and LS diets. N urine excretion tended (*p* = 0.06) to be lower for SF and LS (Table 4), while N excretion in feces was similar (*p* > 0.05) between treatments. Nitrogen balance was similar (4.4 ± 1.8 g N/d) between treatments (*p* > 0.05). There was no negative N balance from dietary treatments, which suggests that the protein intake met the protein requirements of the animals. Although no differences were found in N excretion, it can be observed that N in feces was higher than in urine, which suggests that there was a greater use of ammonia in the rumen, causing a transfer from urine to feces [26].

### 3.3. Milk Composition

No differences in milk yield, FCM 3.5%, protein and NFS were found between treatments (Table 5). Fat content (g/100g) tended to be higher (*p* = 0.09) for control compared to LS. Milk urea N was reduced by SF and LS and this was in line with the observed trend for a reduction in N excretion in urine.

Milk production, milk fat and milk protein responses to SF and LS were consistent with previous studies [4,20,27], supporting the fact that normally oilseeds have no effect on milk yield, enhance milk fat secretion, and induce variable effects on milk protein concentrations in goats. 

In the present study, fat and protein concentrations in milk were higher than those reported by Bernard et al., [20] and Luna et al., [27], when dairy goats were supplemented with SF and LS oils, but similar to Economides, [21] when goats were supplemented with SF meal. Our results point at the fact that the proportion of fiber in the diet was adequate to promote the formation of acetate and butyrate that are the main precursors of the FA synthesized in the mammary gland [5]. This is also supported by the NDF contents found in both SF and LS. Another explanation for the lack of difference in milk fat contents could be a dilution effect, as in this study milk yield was low (around 0.75 kg/d) compared to the cited studies [20,27], which resulted in greater concentrations of fat and protein in milk. 

In this study, milk protein was unaffected by dietary treatments, and one explanation could be that fat supplementation did not affect energy intake, which is one of the most important nutritional factors affecting milk protein [4,28]. For the same reason, NFS were not affected by dietary treatments. Studies that have used oilseed by-products supplementation in goat diets have different milk production traits outcomes, as there is a wide variety of combinations of basal diets (forage and concentrates), and amounts of oilseed by-products in the diets. Our results agree with those studies using a modest amount [29,30] of supplementation enough to supply energy and provoke changes in milk fatty acids contents. 

### 3.4. Milk Fatty Acid Profile

In this study, lipid supplementation did not (*p* > 0.05) influence the concentrations of C4:0 to C14:0 milk FA (g/ 100 g FA) (Table 6). Control increased C16:0 (*p* < 0.001) while LS and SF increased (*p* = 0.01) oleic acid (C18:1). 

Palmitic acid is the main FA in Megalac-R^®^ (control) and is often related to increases in milk fat contents. In goats, feeding C16:0 increases milk C16:0 considerably at the expense of C10:0 to C14:0 and C18:1 [4]. In fact, in this study, content of milk C18:1 was lower (*p* < 0.05) in control. Nudda et al. [30] and Luna et al., [27] did not detect significant changes in the concentration of FA from C6:0 to C12:0 in goat´s milk after supplementation with different amounts of extruded linseed cake or SF respectively. 

In this study, the lack of changes in the contents of short-chain FA (C4:0 to C8:0) was unexpected as they are partially synthesized by metabolic pathways that are dependent of acetyl-CoA carboxylase [31]. Similarly, the decrease of C18:2 observed for LS diet was probably due to the extensive hydrogenation of this acid, abundant in LS and SF, as observed numerically in C18:0 contents in milk. Chilliard and Ferlay, [32] compared the effects of including sunflower oil and oilseeds in goat diets and revealed that oilseed C18:2 was more hydrogenated to C18:0 than oils C18:2. This suggests that the desaturation ratio of stearic acid in the mammary gland is decreased by oil supplemented diets which increase the availability of either PUFA or trans FA, as these FA are putative inhibitors of the delta 9-desaturase [33].

Contrary to the present study, Bernard et al., [20] used plant oils in goat diets resulting in increases (*p* < 0.05) in milk C4:0 and decreases in (*p* < 0.05) C8:0 concentrations. In this study, changes in milk FA composition attributed to SF and LS were characterized by a numerical increase in C18:0 concentration. Those responses are comparable to those reported by Bernard et al., [20] and Luna et al. [27]. Contrary to the present study, in an in vitro study, Zened et al. [34] found an increase in C18:0 when including SF oil compared to diets with starch ranging from 22 to 35% of dietary starch. Our results on C18:0 could be explained by the fact that adding unsaturated FA to the diet cause adaptations of rumen microorganism increasing their ability to hydrogenate unsaturated FA [35].

While C18:2 was reduced in response to LS, C18:3 was not affected by the inclusion of oilseeds (*p* = 0.17). Similarly, decreases in milk C18:2 were reported when goat diets were supplemented with sunflower oil [27] or extruded LS cake [30]. On the contrary, when goats and sheep are fed with linseed [24,36,37], increases in rumenic acid (conjugated linoleic acid; CLA) have been observed, for which the main structure is based on C18:2. In this study, the gas chromatograph program was not able to detect CLA isomers and this is something that future research should take into account as CLA has very high biological value (i.e., cardioprotective) for human health [1]. 

Although, previous studies [38] suggested that when rations are supplemented with linoleic acid rich seeds such as SF, the linoleic acid proportion in milk fat rarely exceeds that observed with un supplemented diets by more than 1.5 %. In this context, Mir et al. [39] added canola oil up to 6 %DM in goat diets and reported no changes in the secretion of linoleic acid in goat’s milk.

Concentrations of total SFA tended to be lower (*p* = 0.08) in SF and LS (Table 6). This was explained by the observed decrease of C16:0 in milk from SF and LS compared to that of control. Total PUFA content was higher (*p* = 0.03) in milk from control, and was consistently lower in milk from animals on LS treatment (Table 5). Nowadays there is a current debate on the effects of dietary SFA on increasing the risk of development of cardiovascular diseases or coronary heart diseases [40]. However, results from the present study suggest that feeding dairy goats with SF or LS is a feasible feeding strategy that tends to produce milk fat with less negative impacts on human health. One dilemma that this study faced is the fact that the inclusion of SF and LS was meant to provoke changes in milk fatty acids without disruptions to the overall animal’s performance. Therefore, if stronger effects on milk fatty acids were sought, it would have been possible to observe negative effects on animal´s productive traits.

## 4. Conclusions

Supplementation with sunflower and linseed whole seeds in grass silage-based diets is a feeding strategy that does not affect nutrients intake and digestibility, milk yield, and milk composition. Moreover, supplementing goat diets with LS or SF can reduce palmitic acid and increase oleic acid, suggesting that both can be effective nutritional strategies for improving milk fatty acid profile from a human health standpoint. 

## Figures and Tables

**Table 1:** Chemical composition (g/kg DM) of individual feedstuffs and lipid supplements used in the experimental diets.

Parameters ^1^	Grass Hay	Grass Silage	Sorghum Grain	Canola Meal	Linseed	Sunflower Seed	Megalac-R^®^
Dry matter	899	279	895	940	945	946	950
Organic matter	907	912	984	900	966	968	908
Crude protein	113	124	83	348	194	153	0
RDP	50	80	21	209	116	122	0
Ether extract	18	30	35	32	270	325	824
Starch	3	10	683	50	100	15	0
NFC	100	248	718	165	0	0	0
NDF	676	510	148	355	586	648	0
ADF	317	284	45	165	349	397	0
ADL	42	43	89	67	213	156	0
Ca	2.5	5	0.5	9	2	3	88
P	2.5	3	5	12	7	12	0
ME(Mcal/kg DM) ^2^	2.1	2.1	3.0	2.6	2.7	2.1	6.0
NEl(Mcal/kg DM) ^2^	1.3	1.3	1.9	1.6	1.7	1.3	5.3
Fatty acids g/100 g fat							
C16:0	23.0	21.0	13.0	6.1	6.6	5.8	48.0
C18:0	3.9	4.0	2.6	1.7	4.4	3.2	5.0
C18:1	12.3	11.0	33.9	62.0	18.5	48.1	36.0
C18:2	15.7	16.8	49.5	24.0	17.2	40.1	9.0
C18:3	32.7	30.7	0.9	5.9	53.2	0.18	0.00

^1^ Dry matter = expressed as fresh matter; RDP = rumen degradable protein; NFC = non-fiber carbohydrates calculated as 100 − (ash + crude protein + ether extract + NDF); NDF = neutral detergent fiber; ADF = acid detergent fiber, ADL = acid detergent lignin; ME = metabolizable energy (Mcal/kg DM); Net energy lactation = NEl (Mcal/kg DM). ^2^ Calculated from NRC [13].

**Table 2:** Chemical composition, nutritional value and fatty acid profile from control (Megalac-R^®^), sunflower (SF) and linseed (LS) treatments (g/kg dry matter (DM).

Components ^1^	Control	Sunflower Seed	Linseed
Dry matter ^‡^	488	495	482
Organic matter	927	933	932
Crude protein	119	112	114
Rumen degradable protein	61.3	58.0	57.9
Ether extract	52.5	46.4	40.6
Starch	212	232	229
Non-fiber carbohydrates	341	352	350
Neural detergent fiber	398	418	417
Acid detergent fiber	196	210	209
Acid detergent lignin	56.1	64.4	65.6
Ca	6.7	3.8	3.8
P	4.0	4.2	3.9
ME, Mcal/kg ^2^	2.48	2.37	2.39
NEL, Mcal/kg ^2^	1.59	1.48	1.49
Fatty acids (g/FA diet)			
C16:0	16.6	5.8	5.7
C18:0	2.1	1.5	1.5
C18:1n 9c	15.5	15.4	8.4
C18:2 n6	10.4	16.1	10.6
C18:3 n3	4.9	4.8	12.1
∑SCFA	18.7	7.4	7.2
∑USFA	30.9	36.2	31.1
∑PUFA	15.3	20.9	22.6

^1^ Dry matter = expressed as fresh matter. Non-fiber carbohydrates were calculated as 100 − (ash + crude protein + ether extract + NDF); ∑SCFA = (C16:0 + C18:0); ∑USFA = (C18:1+C18:2+C18:3); and ∑PUFA = (C18:2 + C18:3). ^2^ Calculated from NRC [13].

**Table 3:** Intake (g/d) and digestibility coefficients (g/kg) in dairy goats fed control (Megalac-R^®^), sunflower (SF) and linseed (LS) treatments (n = 9 per treatment) ^1.^

Parameters ^2^	Control	Sunflower Seed	Linseed	SEM	*p*-Value
Body weight, kg	50.77	50.93	50.60	2.01	0.993
Body weight change, g/d	−50.0	−75.0	−29.2	29.4	0.557
Intake, g/d					
Grass hay	480 ^b^	480 ^b^	509 ^a^	5.66	<0.001
Grass silage	243	246	243	4.99	0.862
Concentrate	558 ^b^	570 ^a^	544 ^c^	0.27	<0.001
Forage:concentrate ratio	(58:42) ^b^	(57:43) ^b^	(59:41) ^a^	0.01	0.001
Dry matter	1281	1297	1298	5.06	0.060
Organic matter	1188 ^b^	1224 ^a^	1197 ^b^	4.62	<0.001
Crude protein	149 ^a^	144 ^b^	143 ^b^	0.62	<0.001
Rumen degradable protein	71.3 ^a^	68.8 ^b^	66.6 ^c^	0.41	<0.001
Ether extract	65.7 ^a^	59.1 ^b^	49.3 ^c^	0.16	<0.001
Neutral detergent fiber	540.7 ^b^	579 ^a^	577 ^a^	2.56	<0.001
Acid detergent fiber	254 ^b^	279 ^a^	276.3 ^a^	1.42	<0.001
Acid detergent lignin	72.3 ^c^	85.2 ^a^	84.3 ^b^	0.23	<0.001
Ca	8.13 ^a^	4.37 ^b^	4.23 ^c^	0.03	<0.001
P	5.03 ^b^	5.50 ^a^	4.83 ^c^	0.02	<0.001
Metabolizable energy, Mcal/ d	3.20 ^a^	3.13 ^b^	3.03 ^c^	0.02	<0.001
Net energy of lactation, Mcal/ d	2.03 ^a^	2.00 ^a^	1.90 ^b^	0.01	<0.001
Fatty acids, g/d					
C16:0	21.3 ^a^	7.07 ^b^	6.83 ^c^	0.04	<0.001
C18:0	2.07 ^a^	1.90 ^b^	1.83 ^c^	0.01	<0.001
C18:1n 9c	20.2 ^a^	20.0 ^b^	10.8 ^c^	0.02	<0.001
C18:2 n6	13.2 ^c^	20.7 ^a^	13.3 ^b^	0.04	<0.001
C18:3 n3	5.37 ^b^	5.37 ^b^	14.9 ^a^	0.04	<0.001
∑SCFA	23.9 ^a^	8.97 ^b^	8.67 ^c^	0.04	<0.001
∑PUFA	38.8 ^a^	14.3 ^b^	14.2 ^b^	0.09	<0.001
Digestibility (g/kg)					
Dry matter	659	637	626	12.6	0.205
Organic matter	689	666	648	11.8	0.075
Neutral detergent fiber	650	617	619	16.6	0.314
Acid detergent fiber	592	545	564	25.1	0.460
Acid detergent lignin	340	410	375	33.6	0.361

^a, b, c^ Different letters indicate significant differences (*p* < 0.05). ^1^ Values are expressed as mean. ∑SCFA = (C16:0 + C18:0) and ∑PUFA = (C18:2 + C18:3). ^2^ Calculated from NRC [13]. SEM = standard error of the mean.

**Table 4:** Nitrogen balance (g N/d) in dairy goats fed control (Megalac-R^®^), sunflower (SF) and linseed (LS) treatments (n = 9 per treatment).

Parameter	Control	Sunflower Seed	Linseed	SEM	*p*-Value
N intake	23.9 ^a^	23.0 ^b^	23.0 ^b^	0.09	<0.001
N excretion					
Feces	10.8	11.1	12.1	0.44	0.119
Urine	8.50	7.16	7.13	0.42	0.062
N balance	4.64	4.69	3.71	0.74	0.584

^a, b^ Different letters indicate significant differences (*p* < 0.05). SEM = standard error of the mean.

**Table 5:** Milk composition from dairy goats fed control (Megalac-R^®^), sunflower (SF) and linseed (LS) treatments (n = 9 per treatment).

Parameters	Control	Sunflower Seed	Linseed	SEM	*p*-Value
Milk yield, kg/d	0.74	0.78	0.77	0.02	0.556
Fat corrected milk 3.5%	0.96	0.99	0.95	0.02	0.656
Milk/DMI	0.57	0.60	0.58	0.03	0.720
Energy corrected milk, kg/d	1.01	1.04	1.01	0.03	0.766
ECM/DMI	0.78	0.80	0.77	0.02	0.724
Milk composition					
Fat, %	5.35	5.14	4.98	0.11	0.086
Fat, g/d	39.9	40.4	38.5	1.68	0.696
True protein, %	4.50	4.55	4.57	0.04	0.609
True protein, g/d	33.6	35.6	35.2	1.24	0.466
Non-fat solids, %	9.65	9.72	9.73	0.05	0.547
Non-fat solids, g/d	71.8	76.0	75.1	2.58	0.486
Milk urea N, mg/dL	28.4 ^a^	26.2 ^b^	25.7 ^c^	0.09	<0.001
Milk-N/N-intake, %	21.9	24.1	24.5	0.83	0.123

^a, b, c^ Mean values for each experiment within a row with unlike superscript letters were significantly different (*p* < 0.05). SEM = standard error of the mean.

**Table 6:** Milk fatty acid profile from goats fed control (Megalac-R^®^), sunflower (SF) and linseed (LS) treatments (n = 9 per treatment) ^1^.

Fatty Acids (g/100g of FA)	Control	Sunflower Seed	Linseed	SEM	*p*-Value
C4:0	1.78	1.27	1.56	0.21	0.27
C6:0	2.70	2.09	2.35	0.25	0.25
C8:0	3.54	2.84	3.07	0.37	0.40
C10:0	11.7	10.1	10.7	1.01	0.52
C12:0	5.09	4.65	4.81	0.35	0.68
C14:0	9.58	11.2	11.4	0.75	0.16
C16:0	28.2 ^a^	23.1 ^b^	22.4 ^b^	0.80	<0.001
C18:0	9.56	10.6	12.0	0.89	0.16
C18:1n 9c	24.1 ^c^	27.5 ^b^	28.3 ^a^	1.03	0.01
C18:2 n6	3.38 ^a^	3.02^ab^	2.45 ^b^	0.20	0.009
C18:3 n3	0.68	0.59	0.75	0.06	0.17
∑Saturated fatty acids ^2^	37.4	33.6	34.4	1.31	0.08
∑Unsaturated fatty acids ^3^	28.1	31.1	31.6	1.34	0.16
∑Polyunsaturated fatty acids ^4^	4.06 ^a^	3.61^ab^	3.21 ^b^	0.22	0.03

^a, b, c^ Different letters indicate significant differences (*p* < 0.05). ^1^ Values are expressed as mean. ^2^ ∑SCFA = (C4:0 to C18:0); ^3^ ∑USFA = (C18:1+C18:2+C18:3); and ^4^ ∑PUFA = (C18:2 + C18:3). SEM = standard error of the mean.
